# Design of image segmentation model based on residual connection and feature fusion

**DOI:** 10.1371/journal.pone.0309434

**Published:** 2024-10-03

**Authors:** Hong Li, Norriza Hussin, Dandan He, Zexun Geng, Shengpu Li

**Affiliations:** 1 School of Information Engineering, Pingdingshan University, Pingdingshan, China; 2 Faculty of Engineering, Built Environment and Information Technology, SEGi University, Kota Damansara, Malaysia; National Textile University, PAKISTAN

## Abstract

With the development of deep learning technology, convolutional neural networks have made great progress in the field of image segmentation. However, for complex scenes and multi-scale target images, the existing technologies are still unable to achieve effective image segmentation. In view of this, an image segmentation model based on residual connection and feature fusion is proposed. The model makes comprehensive use of the deep feature extraction ability of residual connections and the multi-scale feature integration ability of feature fusion. In order to solve the problem of background complexity and information loss in traditional image segmentation, experiments were carried out on two publicly available data sets. The results showed that in the ISPRS Vaihingen dataset and the Caltech UCSD Birds200 dataset, when the model completed the 56th and 84th iterations, respectively, the average accuracy of FRes-MFDNN was the highest, which was 97.89% and 98.24%, respectively. In the ISPRS Vaihingen dataset and the Caltech UCSD Birds200 dataset, when the system model ran to 0.20s and 0.26s, the F1 value of the FRes-MFDNN method was the largest, and the F1 value approached 100% infinitely. The FRes-MFDNN segmented four images in the ISPRS Vaihingen dataset, and the segmentation accuracy of images 1, 2, 3 and 4 were 91.44%, 92.12%, 94.02% and 91.41%, respectively. In practical applications, the MSRF-Net method, LBN-AA-SPN method, ARG-Otsu method, and FRes-MFDNN were used to segment unlabeled bird images. The results showed that the FRes-MFDNN was more complete in details, and the overall effect was significantly better than the other three models. Meanwhile, in ordinary scene images, although there was a certain degree of noise and occlusion, the model still accurately recognized and segmented the main bird images. The results show that compared with the traditional model, after FRes-MFDNN segmentation, the completeness, detail, and spatial continuity of pixels have been significantly improved, making it more suitable for complex scenes.

## 1. Introduction

With the development of Deep Learning (DL) technology, unprecedented breakthroughs have been made in image processing technology. Image Segmentation (IS), as one of the core tasks of computer vision, has been widely used in medical image analysis and visual object tracking [1,2]. In short, IS aims to decompose an image into multiple meaningful regions or objects, laying a solid foundation for subsequent image analysis, recognition, and classification. Although IS technology has made significant progress in recent years, it still faces many challenges, especially in complex backgrounds, multi-objective scenes, and different scale targets [3,4]. At present, the IS is developing rapidly. Traditional IS methods such as threshold-based segmentation, region-based segmentation, and contour-based segmentation techniques are limited. Due to the limited prior knowledge and low-level features, the segmentation effect is often not objective. At this point, Convolutional Neural Network (CNN) technology has emerged, providing new ideas and tools for IS [5]. Among them, the U-Net architecture is widely used due to its efficient processing performance in IS. However, even in DL driven methods, there are still some issues that cannot be fully addressed. For example, when the image content changes significantly or there are significant differences in scale among the targets in the image, traditional CNN structures may be impossible to capture all important contextual information and detailed features. To address the above issues, an IS model based on the residual connection and feature fusion is proposed to improve the training speed and expression ability of the network, making it easier to capture the details and complexity of images.

The content of the article can be mainly divided into four parts. The first mainly exhibits the current research status at home and abroad. The second part elaborates on the FRes-MFDNN. Firstly, an IS model based on cascaded multi-level features is presented, which integrates residual networks to achieve IS. The third is the performance testing and application effect analysis of the model. The fourth summarizes the article.

The main contributions of the experiment are highlighted in two aspects. Firstly, an IS model based on Cascaded Multi-Level Features (CMLF-DNN) is proposed. The core advantage of this model is its ability to fully utilize the deep features of the CNN intermediate layer through the cascade feature fusion strategy. This strategy significantly improves the ability to capture image details, especially when the image content is complex and feature information is rich. Compared with existing models, the CMLF-DNN model significantly improves segmentation accuracy by reducing information loss, especially when dealing with regions with similar textures and colors. Secondly, in view of the multi-scale targets in remote sensing images and many shadows, occlusions, etc., a Full Residual and Multi-scale Feature Fusion (FRes-MFDNN) model is proposed, which adds full residual connections and feature pyramid modules to the convolutional encoding-decoding network. The full residual connection in this model not only simplifies the training process of the model, but also significantly enhances the feature fusion ability. At the same time, the introduced feature pyramid module enables the model to capture contextual information at different scales simultaneously, thereby more effectively responding to changes in target size. Compared with existing models in remote sensing IS tasks, the FRes-MFDNN model demonstrates better robustness and accuracy, especially when target sizes are variable and occlusions exist.

## 2. Related works

IS, as a key task in computer vision, has profound impacts on many practical applications due to its accuracy and efficiency. With the development and application of artificial intelligence algorithms, scholars have begun to analyze the application of deep neural networks in the field of IS. Xia M et al. adopted global attention residual to effectively segment shadows between clouds in satellite images. This method took residual networks as the main framework and introduced an improved route spatial pyramid pool method to extract multi-semantic features at different levels. Experiments on different remote sensing satellite image datasets showed that the research method is significantly superior to existing algorithms [6]. To compare the differences between benign and malignant nodules in cancer patients, Bansal G proposed a cancer segmentation and classification method based on integrated framework Deep3DSCa. During the process, fine-tuning residual networks and morphological techniques were used to extract deep semantic information. The results showed that the segmentation accuracy was 0.927, far exceeding existing methods [7]. Based on DL architecture, Mei and Gül proposed a pixel level crack segmentation method to effectively detect and segment irregular cracks [8]. This method effectively connected the feed-forward neural network of Convolutional Layer (CL) to multiple different layers, and the test results on two data sets showed that the accuracy rate and recall rate of this method were both over 91.00%. Based on deformable contour models for effective segmentation of ultrasound medical image sequences, Ni et al. proposed a DL algorithm. In this method framework, dense residual blocks and attention focused blocks were designed as modules that could effectively propagate lesion areas and applied in practical applications. The results showed that this method exhibited accurate segmentation performance for HIFU ultrasound images [9]. Roy et al. proposed a residual network and compressed excitation network algorithm to effectively address the classification and segmentation of video images. A bi-linear fusion mechanism was also introduced in the design process of the method framework to cope with different types of extrusion operations. The final results demonstrated that this method exhibited superior performance in the IS of the benchmark spectral image dataset [10].

In addition, many scholars have discussed the connection and application between IS technology and DL technology in the medical field. Wu et al. proposed a U-shaped network based on multi-scale attention mechanism to assist in clinical liver treatment and segmentation experiments. The method redesigned the encoder, decoder, and context conversion structure, greatly improving the encoder’s ability to extract features. The results showed that the dice similarity coefficient and joint intersection coefficient of the research method on the public dataset reached 98.00% and 96.08%, respectively [11]. Punn et al. proposed a multi-modal fusion DL method for more effective segmentation of biomedical radiology medical images. This method combined multi-modal fusion, tumor extractor, and tumor segmentation to work together in tumor IS. The results showed that this method performed well in IS [12]. Based on pure dilation residual U-Net, Shen et al. proposed an IS method to segment lower limb bone images. The research method introduced the extended convolution for growing the receptive field of images. Compared with traditional algorithms, the research method had fewer parameters, higher accuracy, and faster system operation efficiency [13]. Based on improved three-dimensional path multi-scale CNN, Meng et al. proposed a segmentation method for human liver and liver tumors to accurately distinguish benign and malignant tumors in the liver. In addition, this study utilized conditional random fields for eliminating erroneous segmentation points. The results showed that when trained on the LiTS common dataset, the research method showed significantly superior performance, with a Dice of 0.965 and a Hausdorff distance of 29.162 for liver segmentation [14]. Punn and Agarwal proposed a computer-aided diagnosis system based on the improved U-Net framework to achieve early diagnosis and treatment of different disease images. During the process, different modules were used to achieve high-precision system models. The research method was validated for image diagnosis of respiratory syndrome virus morphology. The results indicated that the diagnostic efficiency and accuracy of this method were significant, which could promote the development of modern medicine [15]. To accurately segment foam images, Zhong et al. proposed an IS method based on multi-scale attention and generative adversarial networks. This method distinguished images through the discriminator and output a confidence map to determine the parameters of the generator. The results showed that the effectiveness of this method was very obvious, and the accuracy was improved by 5% [16]. To improve the accuracy of medical IS, Xia H et al. proposed a segmentation method based on multi-scale contextual attention mechanism. Image pixels were used as the basis, and weighted calculations were performed on the target image. The results showed that the accuracy, recall rate, AUC value and other indicators were significantly better [17].

In summary, existing IS methods usually achieve image target segmentation by extracting features such as grayscale, color, and texture of the image. When there is a large difference between the target and the background in the image, the image target can be accurately segmented. However, when the difference is small, the segmentation results obtained by existing IS methods often have more errors, resulting in insufficient details in the generated image. At present, the IS is developing rapidly. Residual network and multi-level feature fusion extraction are used in many fields, but there are few studies on its application in the field of IS. To reduce this difference, the experiment proposes an IS method that combines residual connections and feature fusion. Firstly, the CMLF-DNN model is proposed, which enhances the ability to capture complex image details by fusing CNN deep features, and significantly improves segmentation accuracy, especially when processing similar texture and color areas. Secondly, to address the multi-scale and occlusion problems of remote sensing images, the FRes-MFDNN model is designed. This model simplifies training and enhances feature fusion through full residual connection and feature pyramid modules, effectively responds to changes in target size, and improves the accuracy of IS. Stickiness and accuracy.

## 3. Design of is model on the grounds of residual connection and feature fusion

With the development of modern technology, various DL technologies have emerged one after another. Although many DL algorithms have made significant progress in this field, there are still some challenges that have not been fully addressed. How to obtain the necessary information from images and analyze them more accurately has become crucial. To better address this issue, residual connection and feature fusion strategies are introduced in the experiment, aiming to design a more powerful IS model and lay the foundation for future applications.

### 3.1. Construction of is model on the grounds of multi-level features

**Fig 1:**
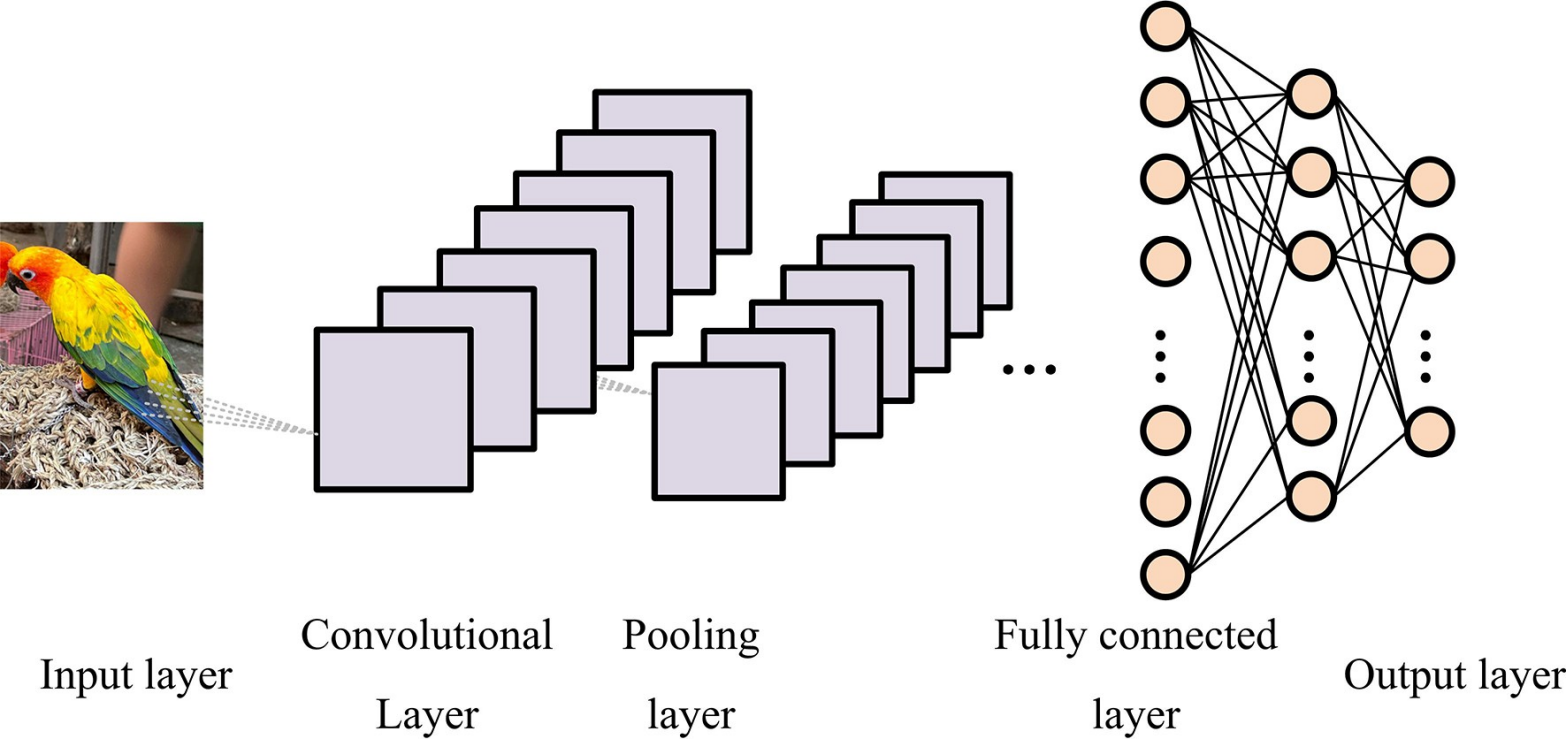
Architecture of CNN model (the image in the figure was originally shot and produced by the author, and does not involve copyright).

Nowadays, DL algorithms are becoming increasingly diverse, especially CNN technology, which has demonstrated outstanding performance in image analysis. The unique advantage of CNN is that it can autonomously extract various features of images from numerous samples through its hierarchical structure. CNN is a feed-forward neural network, specifically used for classification, which usually has multiple layers, such as input layer, Fully Connected Layer (FCL), output layer, and other relevant layers [18,19]. The detailed structure is shown in Fig 1.

In Fig 1, the detailed architecture of CNN can be divided into five layers. The input layer is the two-dimensional image itself, which is a matrix formed by the image pixels. The convolution layer is a feature extraction layer. The convolution operation is the main operation process of the convolution layer. Namely, the convolution kernel is used to slide on the image and multiply the pixel value of the corresponding pixel in the image, and then output after being integrated into the Activation Function (AF). The pooling layer is mainly used to perform pooling operations on feature maps, extracting and retaining useful information based on the principle of local image feature correlation to output smaller feature maps. The FCL is generally located at the end of the CNN, which is used to perform regression classification on the features proposed by all previous layers. The output layer outputs a category probability vector, with each node corresponding to a category. Among them, different CL contains different numbers of convolution kernels, and each convolution kernel extracts a feature of the image and outputs a feature map. The general CL is shown in Eq (1).


$$x_{j}^{l}=f\sum_{i\in M_{j}}\left(x_{i}^{l-1}*k_{ij}^{j}+b_{j}^{l}\right)$$ (1)

In Eq (1), *l* represents the quantity of CL. $k_{ij}^{j}$ represents the convolutional kernel. $b_{j}^{l}$ represents the offset. * represents the convolution operation. *f*(⋅) serves as the AF. Generally, Tanh and Sigmoid functions can be used. $x_{j}^{l}$ is the output of the *j*-th channel in the CL *l*. It is obtained by convolutional summation and bias addition of the previous layer’s output feature map $x_{i}^{l-1}$, and input the AF. *M*_*j*_ serves as the input set of the *j*-th channel, especially for the first CL, which is the pixel value of the image. Next, the image is pooled, with each feature map of the Pooling Layer (PL) uniquely corresponding to the feature map of the previous CL. The pooling operation is shown in Eq (2).


$$x_{j}^{l}=f(\beta_{j}^{l}down(x_{j}^{l-1})+b_{j}^{l})$$ (2)

In Eq (2), $x_{j}^{l}$ represents the output of the *j*-th channel on PL *l*. *down*(⋅) represents the pooling operation. This operation has two advantages. Firstly, the feature dimensionality reduction is performed. Secondly, the scale invariance of feature images can be guaranteed.

The FCL is situated at the end of a CNN, responsible for classifying and processing the features of previous layers. This layer contains a series of neurons, with independent nodes within the layer and tight connections between layers [20]. It combines two-dimensional features into one-dimensional inputs. After weighted synthesis, it is passed into the AF to obtain the output, as showcased in Eq (3).


$$x^{l}=f(w^{l}x^{l-1}+b^{l})$$ (3)

In Eq (3), the activation value of neurons in layer *l* is equal to the activation value of layer *l*−1 multiplied by the weight *w*^*l*^ of layer *l*, and plus the bias. The result is input into the AF *f*(⋅). The FCL is responsible for mapping the convolutional features and PL into category data. Due to the large number of neurons in the FCL, over-fitting easily occurs. In the CNN, the Dropout1 strategy is often used in this layer to invalidate some nodes. After the FCL, the output layer is connected, which is composed of a list of neurons that match the output category. The two layers of neurons are tightly connected, but the nodes within the layer are not connected. The output layer outputs probability vectors for each category, with each node corresponding to a category. The AF is introduced into CL operations, which endows the model with non-linear characteristics and helps the model better overcome the limitations of linear models. The experiment utilizes the Sigmoid function and Tanh function, as demonstrated in Eq (4).


$$\left\{ \begin{array}{l} \sigma (x)=\frac{1}{1+e^{-x}}\\ \sigma^{\prime }(x)=\frac{1-e^{-2x}}{1+e^{-2x}} \end{array}\right.$$ (4)

**Fig 2:**
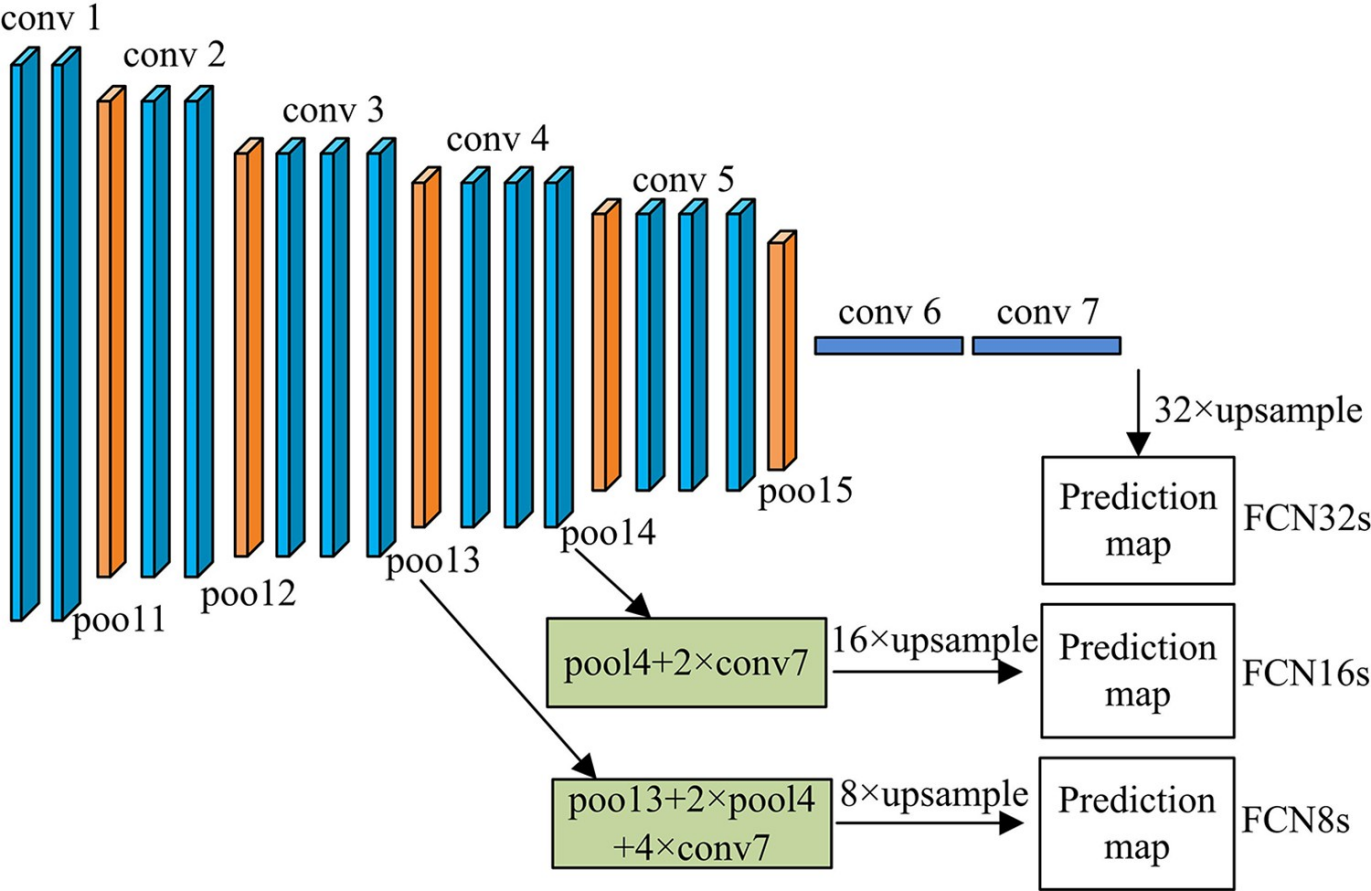
Relevant architecture of FCN8s network model.

In Eq (4), *σ*(*x*) represents the Sigmoid function. *σ*′(*x*) represents the Tanh function. Based on traditional CNN operations, to improve the segmentation ability of CNNs for images, a Fully Convolutional Networks (FCN) is proposed in the experiment to achieve semantic segmentation of images. The relevant architecture is demonstrated in Fig 2.

**Fig 3:**
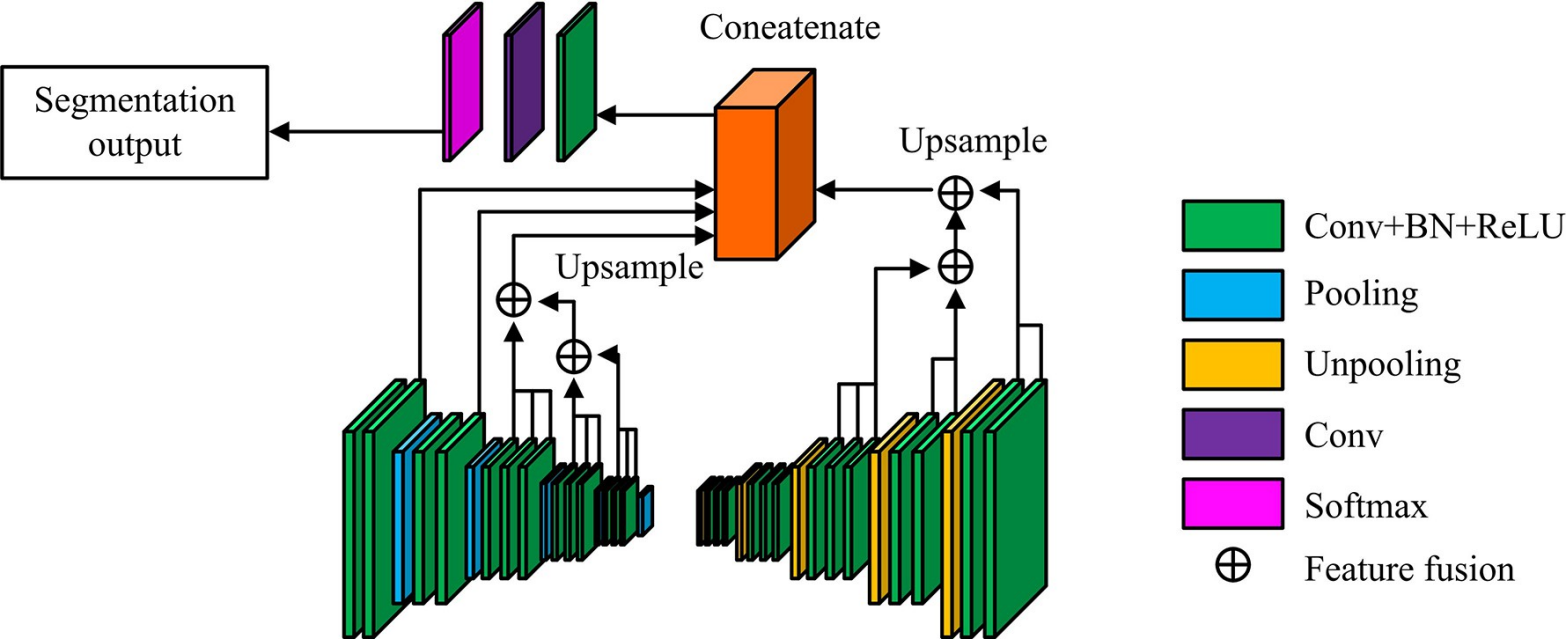
CMLF-DNN model structure (the image in the figure was originally shot and produced by the author, and does not involve copyright).

In Fig 2, the FCN model is migrated from the VGG16 model. By expressing the one-dimensional vector of its FCL into a convolution layer with a convolution kernel size of 1×1, it contains 15 convolution layers and 5 pooling layers. After 5 rounds of pooling, the resolution of the image is reduced by 2, 4, 8, 16, and 32 times. The FCN model up-samples the output feature map of the last layer 32 times to obtain the commonly used FCN32s model. Then the feature maps of different resolutions are up-sampled and fused in a skip structure. The last layer of feature maps is up-sampled 2 times and the results of the fourth pooling layer are fused. The fusion result is up-sampled 16 times to obtain FCN16s. Finally, FCN8s with better effects can be obtained by fusion in this way. FCN transforms the FCL of traditional CNN into a CL, and utilizes de-convolution for expanding the size of feature maps used for pixel level classification, thereby achieving IS. However, multiple pooling and convolution operations in FCN may result in a decrease in the resolution and information loss of the resulting image, ultimately resulting in unclear edges of the segmentation results. Although de-convolution or bi-linear interpolation can restore the feature map size, the lost information cannot be fully retrieved. To alleviate this problem, the experiment considers fusing multi-layer features of CNN, utilizing low and advanced features to enhance IS performance, and reducing the impact of information loss due to repeated pooling and convolution operations on IS performance [21,22]. Therefore, the experiment proposes a Cascaded Multi-level Features Deep Neural Network (CMLF-DNN) model that integrates multi-level features based on CNN encoding and decoding. The structure is illustrated in Fig 3.

In Fig 3, the CMLFDNN model uses the SegNet as the backbone network for segmentation, which contains two components: the encoder and the decoder. The encoder contains 13 CLs and 5 pooling layers to obtain good feature extraction effects. The decoder is a complete mirror image of it, including 13 CLs and 5 depooling layers. Regarding cascaded multi-level features, CMLFDNN integrates all convolutional features and the features of the last three convolutional stages. Specifically, the tail features of conv1 and conv2 in the encoder, as well as the complete features of conv3, conv4, and conv5, are selected. Among them, the features from conv3 to conv5 are extended through bi-linear interpolation and merged pixel-by-pixel [23,24]. In the decoder, the features of the following three stages (conv3_D, conv2_D, conv1_D) are chosen and fused at the pixel level. In summary, after merging channels, a new convolutional and softmax layer is input to output the final segmentation result. The loss function of the model is the cross entropy, as defined in Eq (5).


$$Loss(l,p,\theta )=\frac{1}{N}\sum_{i=1}^{N}\sum_{k=1}^{N}-\sigma (l_{i}=k)\log\;p_{k,i}$$ (5)

In Eq (5), *l*_*i*_ serves as the true label at pixel *i*. *p*_*k*,*i*_ serves as the output probability of pixel *i* belonging to class *k*. *K* represents the total categories. *N* represents the total pixels in the batch image. *σ*(⋅) represents a signed function (when *l*_*i*_ = *k*, it is 1. Otherwise, it is 0). The experiment uses F1 value, Average Precision (AP), and Intersection of Union (IOU) as performance evaluation indicators to estimate the model. The definition of relevant indicators is shown in Eq (6).


$$\left\{ \begin{array}{l} F1=\frac{2*P*R}{P+R}\\ AP=\int_{0}^{1}P(R)dr\\ IOU(P_{m},P_{gt})=\frac{\left|P_{m}\cap P_{gt}\right|}{\left|P_{m}\cup P_{gt}\right|} \end{array}\right.$$ (6)

In Eq (6), *P* = *TP*/(*TP*+*FP*) represents the proportion of correctly predicted positive classes to all. $R=\frac{TP}{TP+FN}$ represents the proportion of correctly predicted positive classes to all samples. *P*_*gt*_ represents the pixel set of the real labeled image. *P*_*m*_ represents the pixel set of the predicted image. ∩ and ∪ serve as intersection and union operations. |⋅| represents the quantity of pixels calculated for this group.

### 3.2. A remote sensing is model integrating full residual connection and feature fusion

**Fig 4:**
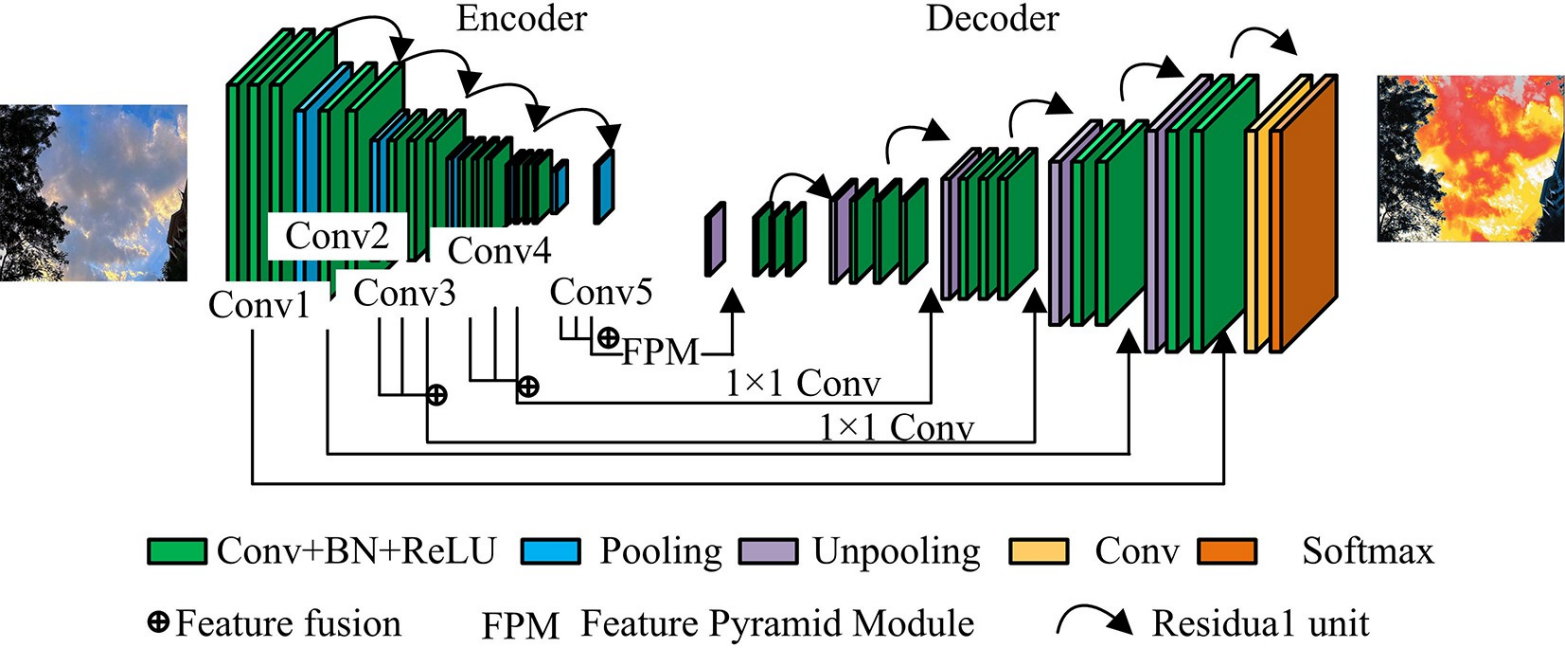
FRes-MFDNN model structure (the image in the figure was originally shot and produced by the author, and does not involve copyright).

In deep CNNs, although multi-scale hollow convolution and spatial pyramid structures can extract multi-scale features, their grid effects and information loss problems affect the final image segmentation quality. In addition, taking deeper CNNs as the backbone network for segmentation can effectively improve segmentation accuracy and overcome gradient vanishing. However, the complex structure significantly increases the memory burden during the training process [25]. To address the above issues, an IS model based on FRes-MFDNN is proposed in the experiment. The architecture of the FRes-MFDNN model is shown in Fig 4.

In Fig 4, the FRes-MFDNN model contains a total of 10 CLs, with the convolutional encoding-decoding network as the backbone network for segmentation. Residual units are used in the conv2, conv3, conv4, and conv5 stages of the encoder and the corresponding stages in the decoder. Simultaneously, the last CL in the conv1 and conv2 stages of the encoder and all CLs in the conv3, conv4, and conv5 stages are extracted. The feature pyramid module is used to extract multi-scale features for the features in the conv5 stage. Finally, the above feature information is integrated into the corresponding layer of the decoder in a pixel-by-pixel addition manner. The FRes-MFDNN model structure has two advantages. Firstly, based on convolutional encoding decoding, the features from the encoder are fused into the decoder, and residual connections for long and short distances are established internally. This connection method can effectively enhance feature fusion and simplify training. Secondly, in the process of fusing the features of the encoder into the decoder, the experiment not only selects shallow features, but also specifically selects deep and advanced features. Then in the fifth stage, it introduces an FPM that can aggregate multi-scale information, which can better handle the scale changes of the target and ultimately effectively improve the segmentation effect.

**Fig 5:**
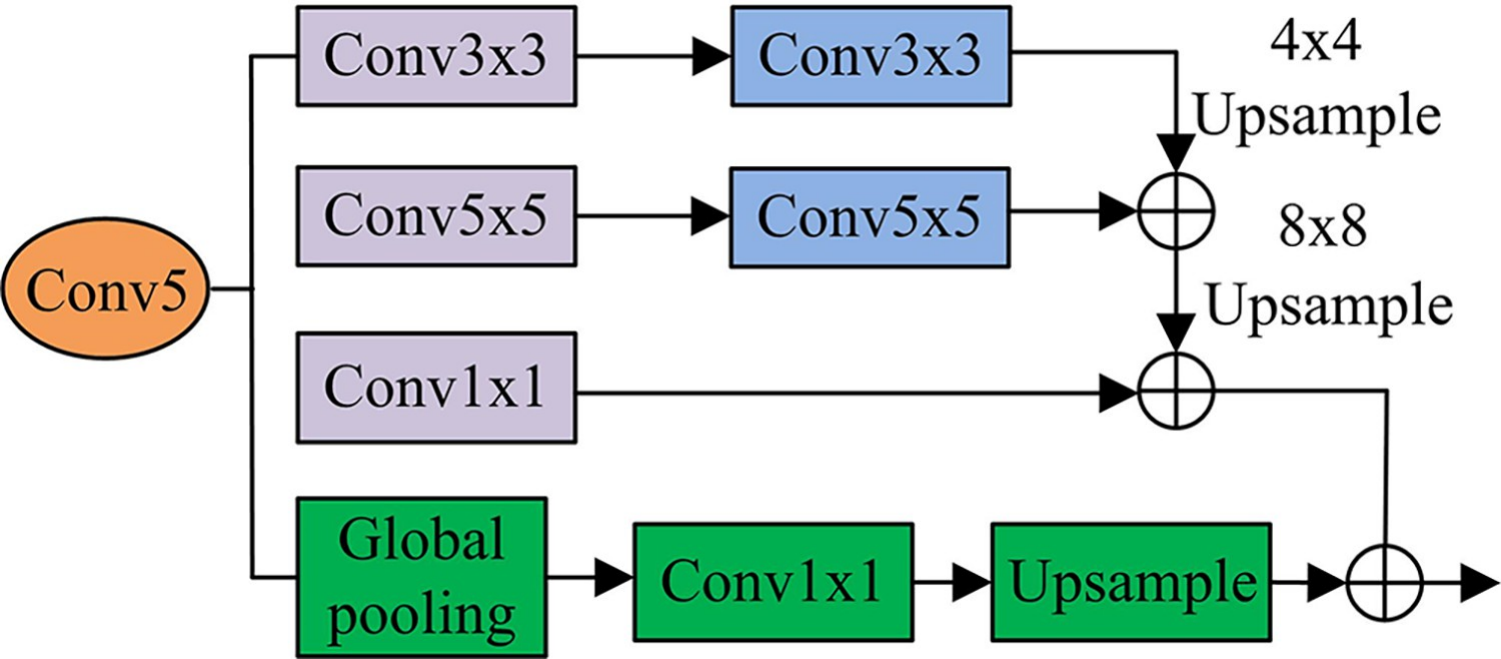
FPM architecture.

Common pyramid structures, such as spatial pyramid pooling in PSPNet and DeepLab, or hollow convolutional modules with ASPP, integrate multi-scale information through parallel channel concatenation. However, this may increase model parameters, and pooling and void convolution may cause information loss or grid effects, which can affect the consistency of feature maps. A new FPM is introduced for this experiment, as shown in Fig 5.

**Fig 6:**
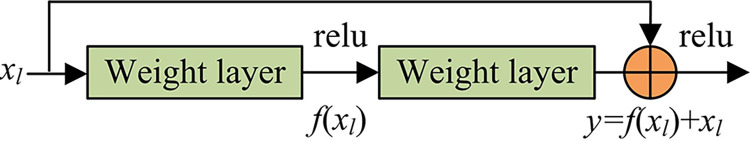
Basic structure of residual network framework.

In Fig 5, the module first extracts the contextual information of the original input feature map (conv5) using convolutions of 3*3 and 5*5, and combines these features to combine adjacent scale contextual features. Next, it performs a 1*1 convolution operation on conv5 and multiplies the results with multi-scale features at the pixel level. Finally, global pooling information is added to effectively enhance the performance of the pyramid module. The FPM runs in the conv5 stage of the FRes-MFDNN model. Due to the low resolution of deep feature maps, using a large convolutional kernel does not increase much computation. Moreover, FPM integrates multi-scale information through pixel-by-pixel addition, comprehensively considering the correlation between features at various scales, and ensuring the consistency of feature information. Meanwhile, to improve the accuracy of traditional CNN models, some scholars have proposed a residual network framework using residual modules. The basic structure is demonstrated in Fig 6.

The residual module in Fig 6 is actually a two-layer network structure, where *x*_*l*_ is the input feature of *l* layer. After weighting and activating the two layers, the mapping function *f*(*x*_*l*_) of *x*_*l*_ can be obtained. Additionally, *y* represents the expected output. The residual is usually represented as *f*(*x*_*l*_) = *y*−*x*_*l*_. The corresponding residual module representation is shown in Eq (7).


$$x_{l+1}=x_{l}+F(x_{l},w_{l})$$ (7)

Then, based on recursion operations, the feature expression form of any layer *L* can be drawn, as shown in Eq (8).


$$x_{L}=x_{l}+\sum_{i=l}^{L-1}F(x_{i},w_{i})$$ (8)

In Eq (8), $\sum_{i=l}^{L-1}F(\cdot )$ serves as the residual. In the back-propagation stage of a neural network, if the LF is assumed to be *E*, then the chain rule based on back-propagation can obtain Eq (9).


$$\frac{\partial E}{\partial x_{l}}=\frac{\partial E}{\partial x_{L}}\frac{\partial x_{L}}{\partial x_{l}}=\frac{\partial E}{\partial x_{L}}(1+\frac{\partial }{\partial x_{l}}\sum_{i=1}^{L-1}F(x_{i},w_{i}))$$ (9)

**Fig 7:**
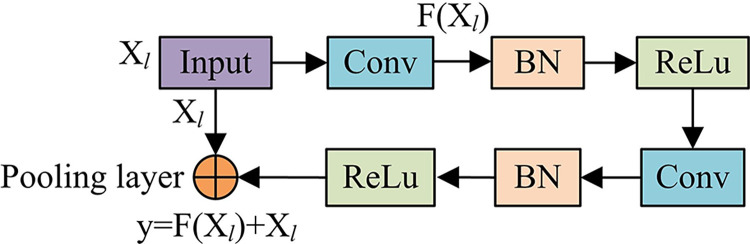
Residual unit structure diagram.

In Eq (9), the first term indicates that the gradient can be directly transmitted back to the shallower layer. The value of the second term $\frac{\partial }{\partial x_{l}}\sum_{i=1}^{L-1}F(x_{i},w_{i})$ cannot be equal to -1. It avoids the gradient vanishing in the network through the above operations. According to the above operations, residual unit blocks are newly constructed in the experiment, with the specific structure shown in Fig 7.

In Fig 7, the residual *F*(*X*_*l*_) is learned from a series of CLs, batch normalization units, and linear correction units. It adds residual units to the convolutional stage corresponding to the encoder and decoder of the FRes-MFDNN model to form a new connection, which is called a short distance residual connection. During the operation, when the system is in the back-propagation stage of the network, the random gradient descent method can be used to update the weights of the entire network, as calculated in Eq (10).


$$Loss(l,p,\theta )=\frac{1}{N}\sum_{i=1}^{N}\sum_{k=1}^{K}-\sigma (l_{i}=k)\log\;p_{k,i}$$ (10)

In Eq (10), *l*_*i*_ serves as the true label at pixel *i*. When *l*_*i*_ = *k*, it is 1. Otherwise, it is 0. *p*_*k*,*i*_ represents the output probability that pixel *i* belongs to class *k*. *K* represents the total categories. *N* represents the total pixels in the batch image. *σ*(⋅) represents a symbolic function. After the above operation, a new segmentation model is obtained. However, the segmentation effect of this model on remote sensing images has obvious defects, which can lead to many issues like multi-scale, occlusion, and shadows in the target to be segmented in the image. To address the above issues, a Conditional Generative Adversarial Network IS model (FMCI-cGAN) integrating multi-scale context information is proposed in the experiment. In this model, assuming that training images *N* are given and their corresponding labeled images are *y*_*n*_, the FMCI-cGAN model obtained is shown in Eq (11).


$$l(\theta_{G},\theta_{D})=\sum_{n=1}^{N}l_{Cross_{G}}(G(x_{n}),y_{n})-\lambda [l_{Cross_{D}}(D(x_{n},y_{n}),1)+l_{Cross_{D}}(D(x_{n},G(x_{n})),0)]$$ (11)

In Eq (11), *θ*_*G*_, *θ*_*D*_ correspond to the parameters of the generated network and the discriminative network. *G*(*x*_*n*_) serves as the generated image of the generated network. The first term represents the LF of the generated network, while the second term represents the LF of the discriminative network. In this model, there are two sub-network structures. For optimizing the entire network structure, the experiment first uses the alternating optimization method to optimize the entire model. The LF corresponding to the discriminative network is showcased in Eq (12).


$$L_{D}=\sum_{n=1}^{N}l_{Cross_{D}}(D(x_{n},y_{n}),1)+l_{Cross_{D}}(D(x_{n},G(x_{n})),0)$$ (12)

In Eq (12), when (*x*_*n*_, *y*_*n*_) is input, the marker of the discriminative network is 1. Otherwise, when (*x*_*n*_,*G*(*x*_*n*_)) is input, the discriminant network’s flag is 0. Next, the LF is calculated, as showcased in Eq (13).


$$L_{G}=\sum_{n=1}^{N}l_{Cross_{G}}(G(x_{n},y_{n})-\lambda l_{Cross_{D}}(D(x_{n},G(x_{n})),0)$$ (13)

In Eq (13), the second term represents the probability that the maximum discriminative network can predict *G*(*x*_*n*_) as the true labeled image of *x*_*n*_, even if the generated image of the generated network is closer to the true labeled image. Finally, the cross entropy function is utilized to represent the LF of the two sub-network structures, as shown in Eq (14).


$$Cross(l,p,\theta )=\frac{1}{N}\sum_{i=1}^{N}\sum_{k=1}^{K}-\sigma (l_{i}=k)\log\;p_{k,i}$$ (14)

## 4. Performance testing and application effects of is models

To comprehensively analyze the specific performance of the constructed model, experiments are conducted to verify the FRes-MFDNN in IS. The experiment is completed on a workstation equipped with Intel(R). The network parameters are initialized using the VGG16 model pre-trained on the ImageNet dataset. The entire model is trained using the Caffe DL framework. An NVIDIA TITANXp memory GPU is used for acceleration during the training process. During training, the model requires that the input original image and label image are three-channel images and spliced into a 600×300 format. The fixed learning rate is 0.0002, batch_size is 3, gamma is 50, momentum is 0.5, and epoch is set to 180 times. Each iteration is 5000 steps, and A is set to 10. During the testing, the test image can be directly input and the segmentation result image can be output. All experiments are conducted in the same environmental conditions. The simulation environment settings are shown in Table 1.

**Table 1:** The experimental basic environmental parameters.

Parameter variables	Parameter selection
Processor	Intel(R) Xeon(R)
CPU	E5-2690 v3
CPU dominant frequency	2.6 GHz
System memory	256 GB
Hard drive	4TB
Training Platform	Caffe Deep Learning Platform
GPU graphics memory	12 GB
Operating system	Windows10
Operating environment	MATLAB
Data storage	MySQL
Data regression analysis platform	SPSS 26.0

The research selects a biomedical segmentation method based on Multi-Scale Residual Fusion structure (MSRF-Net), an IS method based on high-performance semantic segmentation, Atrous convolution and Attention (LBN-AA-SPN), and an adaptive region-based and Otsu-based IS method for comparison. The electronic insulation IS method is compared with the FRes-MFDNN (Adaptive Region Growth-Otsu, ARG—Otsu) [26–28]. To ensure the smooth and fair conduct of the experiment, the four models are run in the same experimental conditions, and all algorithms are set to run the same number of times in the system. Before conducting the formal experiment, the Caltech UCSD Birds200 dataset and ISPRS Vaihingen dataset are selected as task datasets to verify the actual performance of different algorithms.

**Fig 8:**
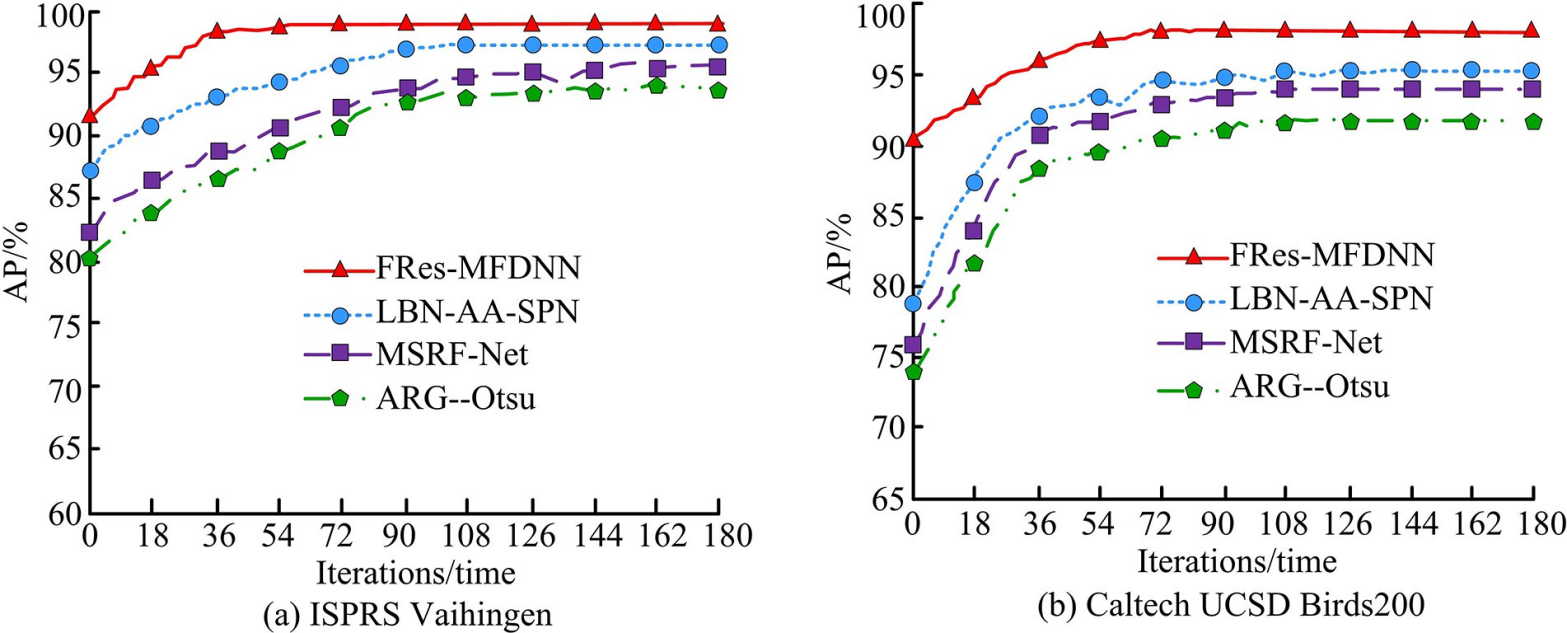
Comparison of AP running on different model.

Firstly, the AP is compared by running four algorithms on two datasets, as shown in Fig 8.

**Fig 9:**
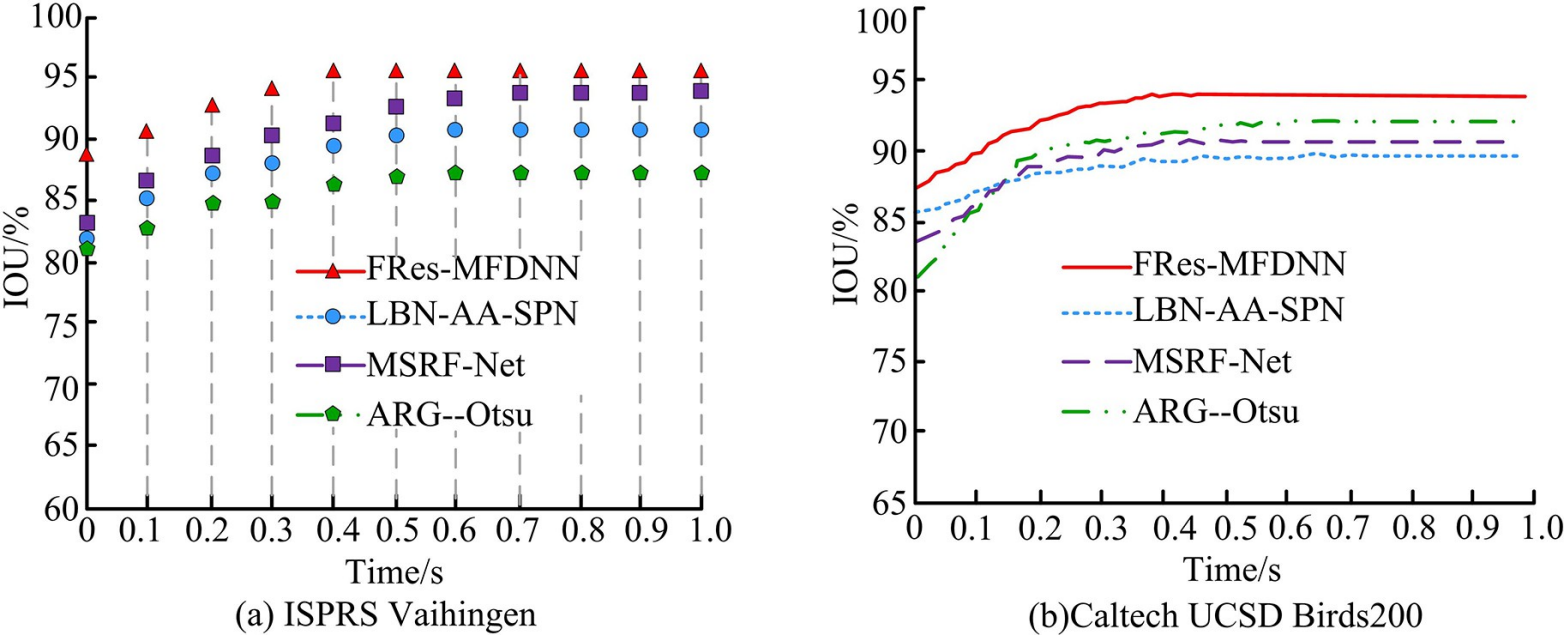
IOU values of four algorithms.

Fig 8A shows the AP obtained by various models running on the ISPRS Vaihingen dataset. As the system iterations increased, the AP has continued to increase. When the number of iterations of the system reached 56, the AP of the FRes-MFDNN was 97.89%. At this time, the AP values of the other three algorithms were still in a constantly changing process. When the system ran 109 times, LBN-AA-SPN had the highest AP value, which was 96.12%. Fig 8B showcases the change test of AP on the Caltech UCSD Birds200 dataset. The initial AP value of the FRes-MFDNN markedly was over 90%. The AP values of other algorithms were all less than 80%. When the system iterated 84, the AP value of the FRes-MFDNN trended towards a stable state, with a value of 98.24%. When the system iterated 126, the three comparison algorithms began to develop towards stability, with a maximum AP value of 95.01%. The AP value of the FRes-MFDNN was markedly higher by 3.23%. The above results all illustrate that the method has higher AP values as well as better segmentation accuracy and performance for images. The IOU value curves are shown in Fig 9.

**Fig 10:**
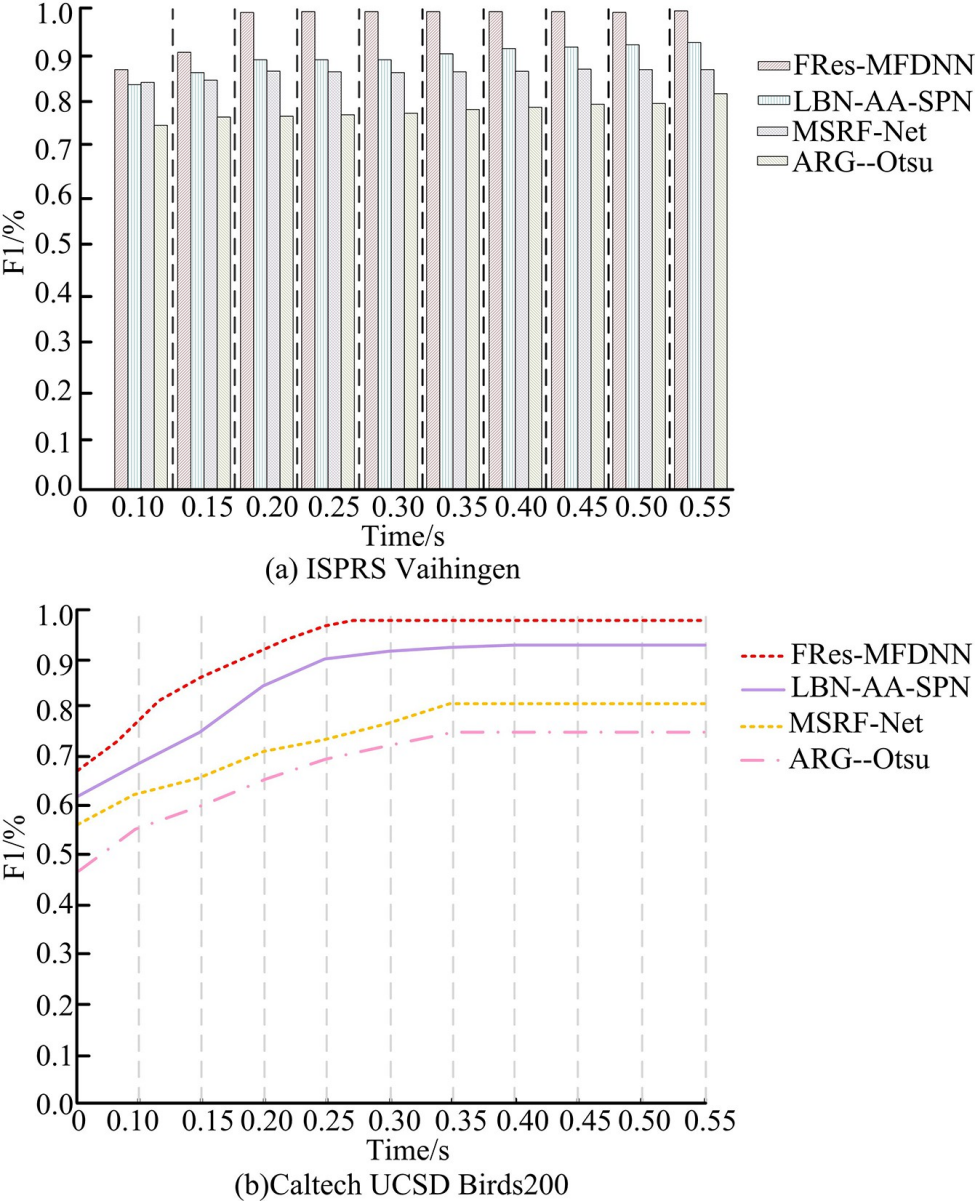
Comparison of F1 values in different datasets.

Fig 9A shows the IOU values of different algorithms in the ISPRS Vaihingen dataset. It demonstrated that the IOU values of all algorithms continued to increase. When the system ran to 0.394s, the FRes-MFDNN had a maximum IOU value of 95.08%. At this point, the IOU values of the three comparative algorithms were all less than 95% and had not reached a stable state. When the approximate system ran to 0.6s, the LBN-AA-SPN algorithm and ARG—Otsu algorithm had stable IOU values. The values reached 91.32% and 88.79% respectively. The IOU value of the MSRF-Net was continuously increasing, approaching but always smaller than the FRes-MFDNN. Fig 9A shows the IOU value variation curves of four algorithms on the Caltech UCSD Birds200 dataset. When the IOU value of the FRes-MFDNN reached its maximum value, the operating time of the system was 0.416s, and the corresponding IOU value was 94.38%. At this point, the IOU values of the LBN-AA-SPN algorithm, MSRF-Net algorithm, and ARG—Otsu algorithm were all less than 94.0%. It is shown that the IOU of the FRes-MFDNN always maintains the highest value in the continuous operation of the system. The high prediction accuracy of IS results in better segmentation performance. Based on the above results, the study compares the F1 values on the two datasets, as shown in Fig 10.

**Fig 11:**
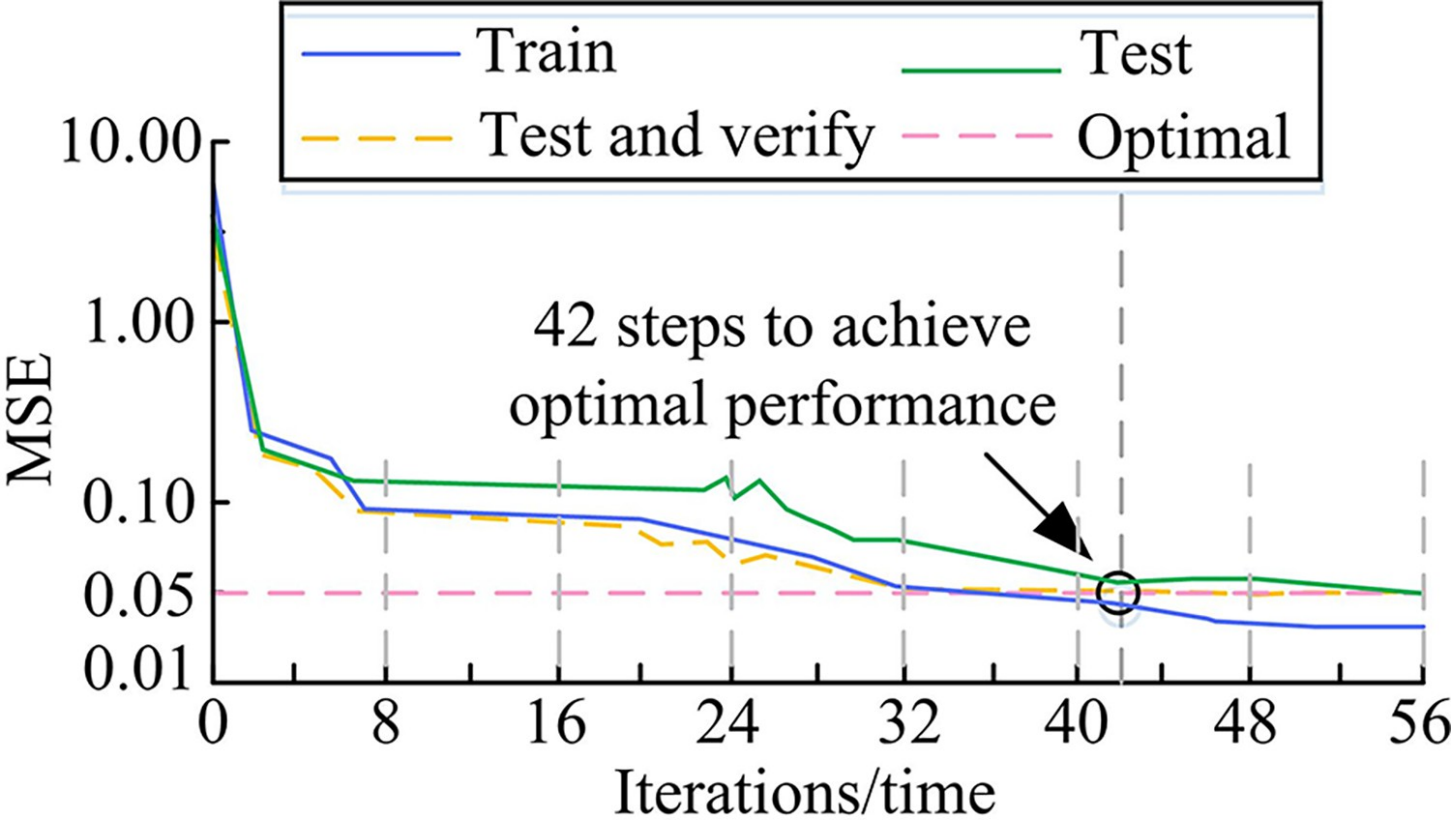
Error results of IS.

Fig 10A shows the F1 values on the ISPRS Vaihingen dataset for different datasets. It demonstrates that as the system running time grows, the F1 values of all four algorithms show a clear upward trend. Compared with other comparison algorithms, the FRes-MFDNN showed higher F1 value. When the running time reached 0.20s, the FRes-MFDNN had the maximum F1 value. Since then, it has been maintained at this value, with an F1 value of 0.999 and infinitely approaching 100%. The F1 values of other methods were smaller than those of the FRes-MFDNNs. Fig 10B shows the F1 value changes of four algorithms on the Caltech UCSD Birds200 dataset. When the F1 value of the FRes-MFDNN approached 100% infinitely, the corresponding system running time was 0.26s. The F1 values of the LBN-AA-SPN algorithm, MSRF-Net algorithm, and ARG—Otsu algorithm were 0.920, 0.773, and 0.717. This indicates that a significant difference exists in the F1 value between the FRes-MFDNN and others, demonstrating that the FRes-MFDNN has small errors in IS and measurement, with better performance. 70% of the Caltech UCSD Birds200 dataset is selected as the training set, 30% of the ISPRS Vaihingen dataset is selected as the validation set, and the remaining two datasets are selected as the testing set for correlation analysis. The Mean Square Error (MSE) results of the FRes-MFDNN are compared, as shown in Fig 11.

**Fig 12:**
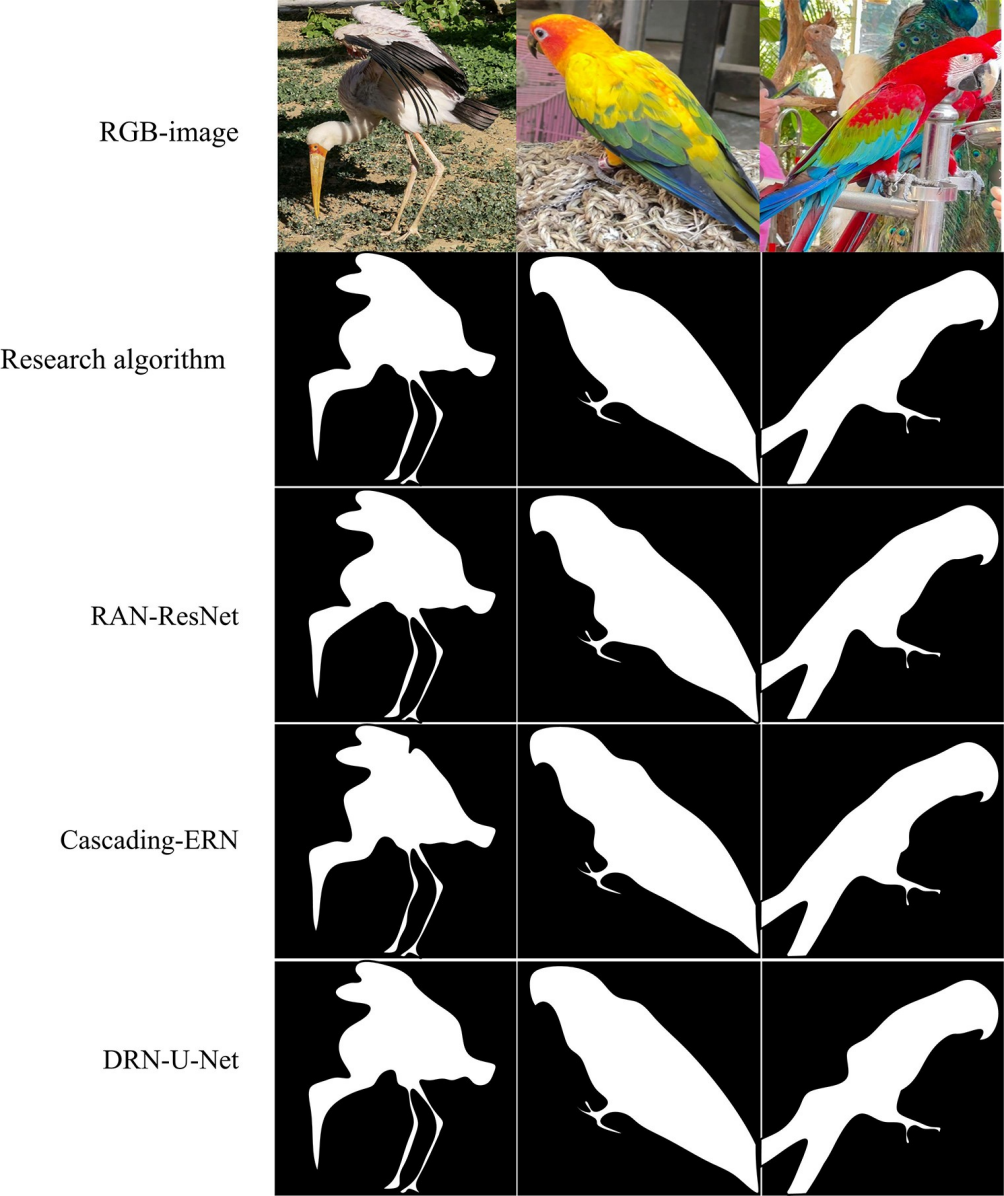
The segmentation effect of four models in unlabeled bird images (the image in the figure was originally shot and produced by the author, and does not involve copyright).

Fig 11 demonstrated that when the quantity of system training iterations increased, the MSE of the FRes-MFDNN showed a cliff like decrease and infinitely approached the target value. When the system iteration reached the 42nd, the MSE value of the proposed model for IS approached the target value of 0.05, which was very close to the target value required by the experiment, achieving optimal performance. Next, the robustness of the model was verified. Different types of bird images were downloaded from the Baidu website library in China, and all images were color RGB images. They were uniformly processed to a size of 256*256, and the processed images were input into the selected model for testing. Some results are shown in Fig 12.

**Fig 13:**
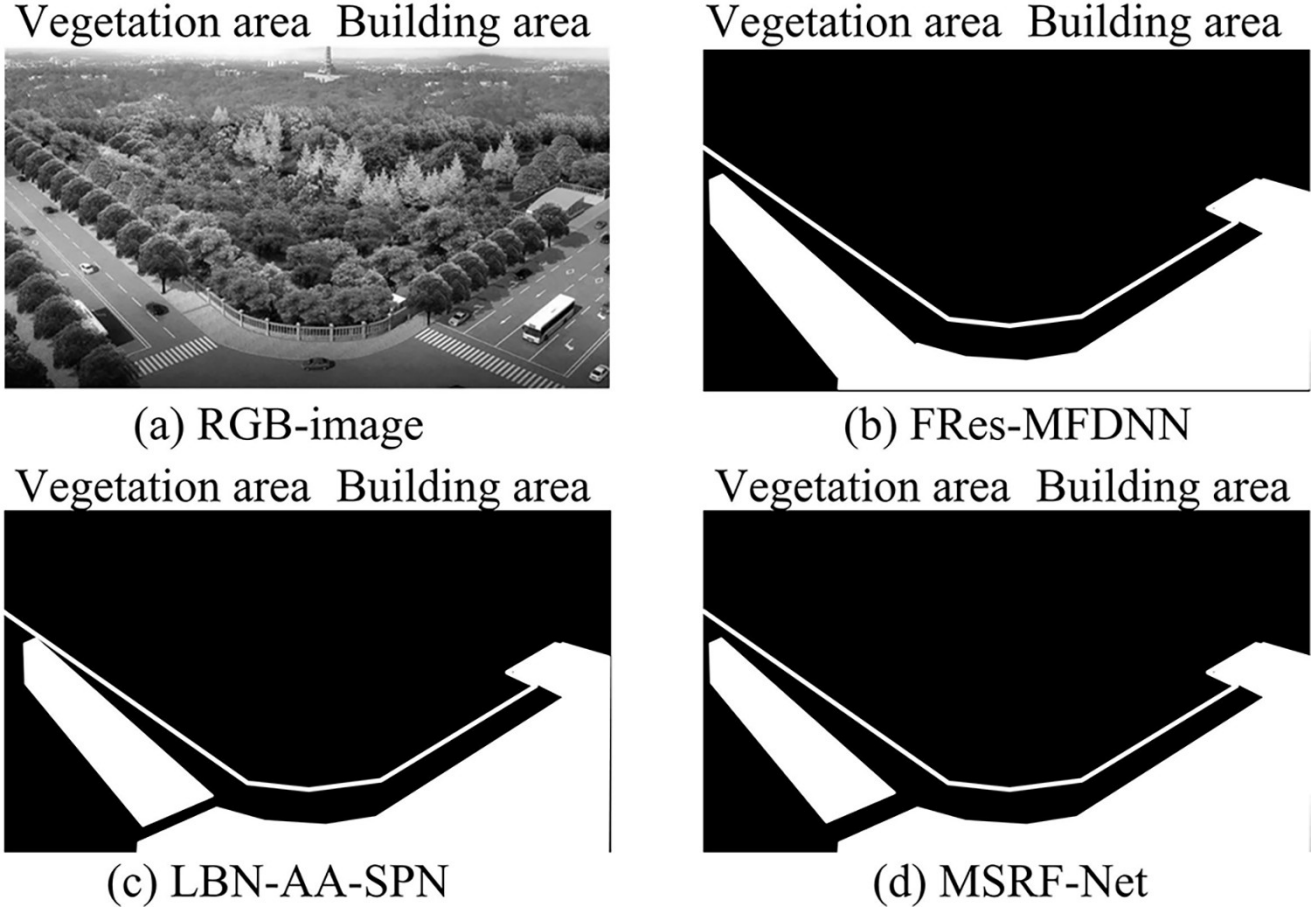
The segmentation effect of different algorithms on building objects and vegetation (the photos were taken by the author and there are no copyright issues).

The types, colors, and postures of birds in Fig 12 vary, with one image containing two targets and partially overlapping. The results in the above Figure indicate that the segmentation results of the research model can generally segment all the birds in the figure, with some details relatively complete, as well as the overall influence is more excellent than the segmentation results of the other three comparison models. This fully demonstrates that integrating different levels of features can provide more contextual information for final prediction, and the overall generalization performance of the model is excellent. At the same time, in ordinary scene images, despite noise and occlusion, the model is still able to accurately identify and segment the main bird images. Then the segmentation effects of the LBN-AA-SPN algorithm, MSRF-Net algorithm, and FRes-MFDNN on building images are compared. The input image size of all models is 256*256, and they are all color images. The specific comparison results are shown in Fig 13.

In Fig 13, the sizes of the targets in each image are significantly different, with varying shapes and degrees of shadow occlusion. Although the building location could be roughly determined, the preservation of edge details was incomplete. In addition, the FRes-MFDNN could more accurately determine the building location and preserve complete edge details. This indicates that the model can effectively fuse features of different scales, which is particularly evident when segmenting images of different sizes. From large buildings to vegetation, the model delivers accurate segmentation results. The segmentation results of vegetation in the image show the sensitivity of the model to details, and even smaller objects can be accurately identified and segmented. The segmentation results (Precision) and experimental time of different algorithms running on the ISPRS Vaihingen dataset are compared, and the specific results are shown in Table 2.

**Table 2:** Segmentation results and experimental time of different models.

Model	Precision/%				T/s
	Image 1	Image 2	Image 3	Image 4	
ARG—Otsu	80.85	84.72	84.88	85.84	37
MSRF-Net	88.26	87.37	89.15	88.48	35
LBN-AA-SPN	89.23	89.47	91.19	89.58	31
FRes-MFDNN	91.44*	92.12*	94.02**	91.41*	28**
CMLF-DNN	87.21	85.36	87.48	87.89	36
FRes	80.84	84.22	85.12	84.08	38

Note

"*" means that there is a significant difference between the results calculated by the FRes-MFDNN and the results of other methods, (*P*<0.05).

"**" means that there is a very significant difference among different results, (*P*<0.01).

From Table 2, the segmentation precision of the the FRes-MFDNN on images 1, 2, 3, and 4 was 91.44%, 92.12%, 94.02% and 91.41% respectively. The segmentation precision of the other comparison methods always was smaller than the FRes-MFDNN. Taking RAN-ResNet as an example, its segmentation precision on images 1, 2, 3, and 4 was 89.23%, 89.47%, 91.19% and 89.58% respectively. In addition, the CMLF-DNN and FRes-MFDNN models were separately applied to four different images for ablation experiments. The data showed that the segmentation precision of CMLF-DNN on images 1, 2, 3, and 4 were 87.21%, 85.36%, 87.48%, and 87.89%, respectively. The time consumption was less than ARG-Otsu, while the IS accuracy of the FRes-MFDNN model was not high. Four images was significantly higher than 90%. There was a significant statistical difference between the IS results of this FRes-MFDNN and other methods. This indicated that the IS precision of the method constructed in the experiment is relatively significant. In summary, It can be seen that FRes-MFDNN has superior performance and higher precision in segmenting edge images.

## 5. Conclusion

After in-depth research and multiple experiments, the study successfully designed an IS model based on residual connection and feature fusion. After introducing residual networks to improve the training stability of the system and integrating features from different levels, the segmentation effect in multi-scale scenes was ensured. From the data results, in the Caltech UCSD Birds200 dataset, when the system was run 84 times, the AP value of the FRes-MFDNN was 98.24% and trended towards a stable state. In the comparison of IOU values, in the ISPRS Vaihingen dataset, when the system ran to 0.394s, the FRes-MFDNN had the maximum IOU value, which was 95.08%. In the comparison of F1 values, on the Caltech UCSD Birds200 dataset, when the F1 value approached 100% infinitely, the corresponding running time was 0.26s. The F1 values of the LBN-AA-SPN algorithm, MSRF-Net algorithm, and ARG—Otsu algorithm were 0.920, 0.773, and 0.717, respectively. When segmenting an unmarked bird image, it was found that the FRes-MFDNN could generally segment all the birds in the image, with some details relatively complete. The overall influence was more excellent than the segmentation results of the other three comparison models. By segmenting the original image in the ISPRS Vaihingen dataset, it was found that the segmentation results of the FRes-MFDNN were significantly superior to other algorithms, which was very close to the label results. The above results indicates that the FRes-MFDNN has high feasibility in segmenting remote sensing images, significantly reduces the error rate of IS results, and has good applicability. Although the model performs well in most cases, it often introduces many new weight parameters to the model when implementing feature fusion, such as the number of convolutional layers and pooling layers, resulting in a slower model training speed. Therefore, future work can consider new feature fusion methods or fusion paths, such as end-to-end feature fusion network and multi-modal feature fusion, etc., to reduce the weight scale, further optimize the model structure, and explore more feature fusion strategies. At the same time, this method is applied to other computer vision tasks to verify its broad applicability and explore more possibilities.

## Supporting information

S1 Dataset (DOC)
