# Supplementary material for: Design of image segmentation model based on residual connection and feature fusion
Source: PLoS One. 2024 Oct 3;19(10):e0309434. doi: 10.1371/journal.pone.0309434 (PMC11449362; doi:10.1371/journal.pone.0309434)
Supplement: S1 Dataset — (DOC) [file pone.0309434.s001.doc]

**The data in Figure 8**

| Epoch | ISPRS Vaihingen-AP(%) | | | |
| --- | --- | --- | --- | --- |
|  | Research algorithm | LBN-AA-SPN | MSRF-Net | ARG--Otsu |
| 0 | 91.64 | 87.56 | 82.07 | 80.01 |
| 18 | 95.01 | 91.21 | 86.14 | 84.03 |
| 36 | 98.78 | 92.49 | 87.55 | 86.14 |
| 54 | 98.79 | 93.72 | 89.91 | 87.55 |
| 72 | 98.99 | 92.26 | 91.94 | 90.04 |
| 90 | 98.99 | 95.54 | 93.87 | 92.51 |
| 108 | 98.99 | 96.11 | 93.98 | 93.07 |
| 126 | 98.99 | 96.12 | 93.98 | 93.16 |
| 144 | 98.99 | 96.12 | 93.98 | 93.16 |
| 162 | 98.99 | 96.12 | 93.98 | 93.16 |
| 180 | 98.99 | 96.12 | 93.98 | 93.16 |
| Epoch | Caltech-UCSD Birds200-AP(%) | | | |
|  | Research algorithm | LBN-AA-SPN | MSRF-Net | ARG--Otsu |
| 0 | 90.11 | 78.45 | 76.21 | 74.12 |
| 18 | 92.74 | 86.79 | 84.47 | 82.13 |
| 36 | 96.02 | 92.13 | 91.87 | 86.78 |
| 54 | 97.48 | 93.79 | 92.08 | 88.45 |
| 72 | 97.71 | 94.18 | 92.77 | 89.87 |
| 90 | 98.24 | 94.20 | 92.81 | 91.12 |
| 108 | 98.24 | 94.99 | 94.02 | 91.77 |
| 126 | 98.24 | 95.01 | 94.02 | 91.77 |
| 144 | 98.24 | 95.01 | 94.02 | 91.77 |
| 162 | 98.24 | 95.01 | 94.02 | 91.77 |
| 180 | 98.24 | 95.01 | 94.02 | 91.77 |

**The data in Figure 9**

| Time/s | ISPRS Vaihingen-IOU(%) | | | |
| --- | --- | --- | --- | --- |
|  | Research algorithm | LBN-AA-SPN | MSRF-Net | ARG--Otsu |
| 0 | 88.77 | 82.03 | 83.54 | 81.42 |
| 0.1 | 90.02 | 85.01 | 85.12 | 82.51 |
| 0.2 | 92.51 | 87.11 | 87.21 | 84.16 |
| 0.3 | 94.03 | 88.21 | 90.01 | 84.16 |
| 0.4 | 95.08 | 89.03 | 91.24 | 86.04 |
| 0.5 | 95.08 | 89.94 | 92.51 | 87.07 |
| 0.6 | 95.08 | 91.32 | 93.33 | 88.79 |
| 0.7 | 95.08 | 91.32 | 94.04 | 88.79 |
| 0.8 | 95.08 | 91.32 | 94.04 | 88.79 |
| 0.9 | 95.08 | 91.32 | 94.04 | 88.79 |
| 1.0 | 95.08 | 91.32 | 94.04 | 88.79 |
| Time/s | Caltech-UCSD Birds200-IOU(%) | | | |
|  | Research algorithm | LBN-AA-SPN | MSRF-Net | ARG--Otsu |
| 0 | 87.52 | 85.22 | 84.01 | 81.89 |
| 0.1 | 89.21 | 86.34 | 86.32 | 86.33 |
| 0.2 | 92.21 | 87.31 | 89.08 | 89.12 |
| 0.3 | 93.58 | 88.60 | 89.11 | 91.48 |
| 0.4 | 94.33 | 89.13 | 91.48 | 91.92 |
| 0.5 | 94.38 | 89.13 | 91.48 | 92.21 |
| 0.6 | 94.38 | 89.13 | 91.48 | 92.21 |
| 0.7 | 94.38 | 89.13 | 91.48 | 92.21 |
| 0.8 | 94.38 | 89.13 | 91.48 | 92.21 |
| 0.9 | 94.38 | 89.13 | 91.48 | 92.21 |
| 1.0 | 94.38 | 89.13 | 91.48 | 92.21 |

**The data in Figure 10**

| Time/s | ISPRS Vaihingen-F1(%) | | | |
| --- | --- | --- | --- | --- |
|  | Research algorithm | LBN-AA-SPN | MSRF-Net | ARG--Otsu |
| 0.00 | / | | | |
| 0.10 | 0.885 | 0.861 | 0.848 | 0.744 |
| 0.15 | 0.912 | 0875 | 0.850 | 0.768 |
| 0.20 | 0.999 | 0.889 | 0.875 | 0.768 |
| 0.25 | 0.999 | 0.889 | 0.875 | 0.771 |
| 0.30 | 0.999 | 0.889 | 0.875 | 0.775 |
| 0.35 | 0.999 | 0.901 | 0.875 | 0.780 |
| 0.40 | 0.999 | 0.911 | 0.875 | 0.782 |
| 0.45 | 0.999 | 0.911 | 0.878 | 0.793 |
| 0.50 | 0.999 | 0.915 | 0.878 | 0.793 |
| 0.55 | 0.999 | 0.920 | 0.878 | 0.811 |
| Time/s | Caltech-UCSD Birds200n-F1(%) | | | |
|  | Research algorithm | LBN-AA-SPN | MSRF-Net | ARG--Otsu |
| 0.00 | 0.679 | 0.616 | 0.565 | 0.477 |
| 0.10 | 0.780 | 0.689 | 0.618 | 0.568 |
| 0.15 | 0.864 | 0.750 | 0.651 | 0.600 |
| 0.20 | 0.911 | 0.848 | 0.705 | 0.650 |
| 0.25 | 0.977 | 0.901 | 0.714 | 0.699 |
| 0.30 | 0.989 | 0.904 0.920 | 0.7230.773 | 0.6850.717 |
| 0.35 | 0.989 | 0.924 | 0.773 | 0.724 |
| 0.40 | 0.989 | 0.924 | 0.773 | 0.724 |
| 0.45 | 0.989 | 0.924 | 0.773 | 0.724 |
| 0.50 | 0.989 | 0.924 | 0.773 | 0.724 |
| 0.55 | 0.989 | 0.924 | 0.773 | 0.724 |

**The data in Figure 11**

| Iterations/time | MSE | | | |
| --- | --- | --- | --- | --- |
|  | Train | Test and verify | Test | Optimal |
| 0 | 8.00 | 6.00 | 6.00 | 0.05 |
| 8 | 0.901 | 0.900 | 0.13 |  |
| 16 | 0.899 | 0.888 | 0.11 |  |
| 24 | 0.078 | 0.071 | 0.10 |  |
| 32 | 0.051 | 0.05 | 0.075 |  |
| 40 | <0.05 |  | 0.060 |  |
| 48 |  |  | 0.055 |  |
| 56 |  |  | 0.050 |  |
